# Regulation of Oocyte Apoptosis: A View from Gene Knockout Mice

**DOI:** 10.3390/ijms24021345

**Published:** 2023-01-10

**Authors:** Sandeep Kaur, Manabu Kurokawa

**Affiliations:** Department of Biological Sciences, Kent State University, Room 108, Kent, OH 44242, USA

**Keywords:** Bcl-2, BH3-only proteins, caspase, cell death, DNA damage, p53, p63, p73

## Abstract

Apoptosis is a form of programmed cell death that plays a critical role in cellular homeostasis and development, including in the ovarian reserve. In humans, hundreds of thousands of oocytes are produced in the fetal ovary. However, the majority die by apoptosis before birth. After puberty, primordial follicles develop into mature follicles. While only a large dominant follicle is selected to ovulate, smaller ones undergo apoptosis. Despite numerous studies, the mechanism of oocyte death at the molecular level remains elusive. Over the last two and a half decades, many knockout mouse models disrupting key genes in the apoptosis pathway have been generated. In this review, we highlight some of the phenotypes and discuss distinct and overlapping roles of the apoptosis regulators in oocyte death and survival. We also review how the transcription factor p63 and its family members may trigger oocyte apoptosis in response to DNA damage.

## 1. Introduction

**Figure 1:**
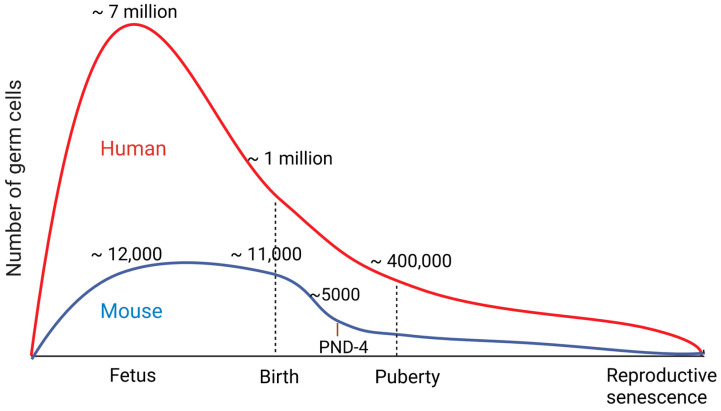
Graphical representation of germ cell quantities in mouse and human ovaries at various stages of the reproductive lifespan. Germ cells are most abundant during the fetal stage. In mice, the greatest loss is seen within the first few days following birth, whereas in humans, substantial germ cell death takes place before birth. Between birth and puberty, there is a second wave of significant decline in the number of oocytes. Once the oocytes are exhausted, the ovaries enter reproductive senescence. PND: Postnatal day.

In the fetal stage, mammalian ovaries contain millions of primordial germ cells. However, more than two-thirds of them are lost prior to birth in humans (or within the first days after birth in mice) [1,2,3]. This number gradually drops as oogonia enter their first meiotic division and become primary oocytes during the period after birth and before puberty (Figure 1). The primary oocytes are maintained in this dormant, meiotically arrested stage (the diplotene stage of the first meiotic prophase, also commonly referred to as the germinal vesicle (GV)) until the luteinizing hormone (LH) surge takes place during puberty. Each oocyte is enclosed by a single layer of epithelial granulosa cells as a primary oocyte (primordial follicle). During an estrus cycle, a group of primordial follicles grows to their maximum size, while the follicle continues to increase the number of granulosa cells surrounding an oocyte. Depending on the number of granulosa cell layers and the size, follicles are categorized as either primary, secondary, pre-antral, or antral. An antral follicle is fully developed and consists of a large oocyte surrounded by several layers of granulosa cells. In response to the LH surge, the oocyte resumes meiosis. The nuclear membrane of the GV breaks down (germinal vesicle breakdown, GVBD) and the first metaphase of meiosis (MI) is completed. Afterwards, the oocyte is arrested again at the second metaphase of meiosis (MII), which is released into the oviduct (ovulation). Importantly, at each estrus cycle, the majority of the “recruited” oocytes die by apoptosis (follicular atresia), while only select oocytes ovulate. Over time, the number of available oocytes per ovary (the ovarian reserve) continues to decline after each estrus cycle until it depletes, which is called reproductive senescence or menopause (Figure 1). Here, we oversee various gene knockout mice generated to date that characterize how each apoptotic player might contribute to the loss of female germ cells in vivo.

## 2. Apoptosis Pathways

**Figure 2:**
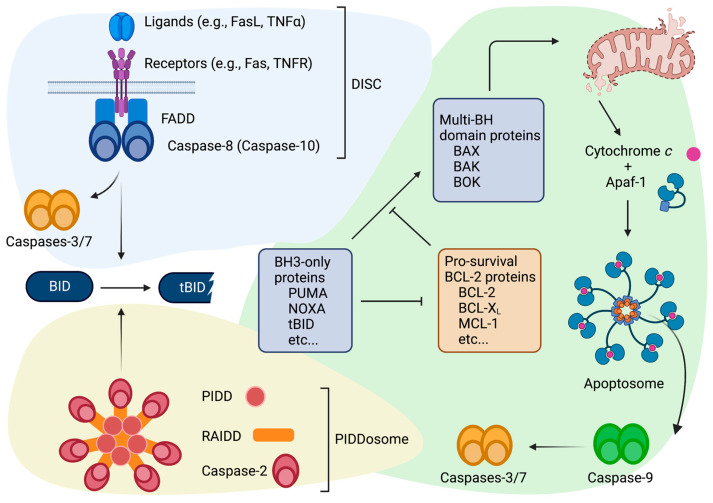
Schematic diagram of the major players in the apoptotic pathways. Three signaling pathways in cells can result in apoptosis. Extracellular stress signals activate the death receptors, which leads to the formation of the DISC complex. Caspases-8 and -10 are then activated by the DISC through dimerization. The second pathway is triggered by an intracellular stress stimulus leading to mitochondrial cytochrome *c* release which is governed by the BCL-2 family proteins. In the cytoplasm, cytochrome *c* binds to the adaptor protein Apaf-1, which triggers the formation of the caspase-9 activating complex apoptosome. The executioner caspase-3 and -7 are subsequently activated via the proteolytic cleavage by caspase-8 (-10) or caspase-9. The third pathway, which is also triggered by an intracellular signal, results in PIDDosome formation and subsequent activation of the initiator caspase-2. The activation of BID by caspase-2 or caspase-8 (-10) feeds into the BCL-2 family-mediated cytochrome *c* release.

Apoptosis is a form of programmed cell death defined by the activation of a group of cysteine proteases called caspases (see [4,5,6] for review). While caspases are kept in a zymogenic form under non-stressed conditions, they are activated in response to various genotoxic or cytotoxic signals. Once active, caspases cleave their specific cellular substrates, leading to the ultimate dismantling of the cell. There are two main pathways to initiate downstream caspase activation: the extrinsic and intrinsic pathways (Figure 2). The extrinsic pathway is characterized by the activation of a cell death receptor (e.g., Fas and TNFR) by its ligand (e.g., FasL and TNFα), which results in the activation of caspase-8 or -10 through the formation of the DISC (death-inducing signaling complex). On the other hand, the intrinsic pathway involves mitochondrial cytochrome *c* release, which is regulated by the BH domain-containing BCL-2 protein family. This broad family consists of both pro-apoptotic (see below) and pro-survival (anti-apoptotic) members (BCL-2, BCL-XL, BCL-W, MCL-1 and A1/BFL-1). The pro-apoptotic members are further divided into two categories based on their structures: the BH3-only proteins and the multi-BH domain proteins. BAX, BAK, and BOK are among the multi-BH domain proteins that contain the BH1, BH2, and BH3 domains. The BH3-only proteins (BAD, BIK, BID, PUMA, BIM, BMF, NOXA, and HRK) only contain a single BH3 domain. Following a cell death stimulus, the expression of one or more BH3-only proteins is induced, which directly or indirectly activates multi-BH domain pro-apoptotic species. This process is counteracted by pro-survival BCL-2 family proteins. Thus, the activation of the multi-BH domain proteins is determined by the balance between the pro-survival BCL-2 family members and the BH3-only proteins (Figure 2). Once activated, the multi-BH domain proteins form a homo-oligomer on the outer mitochondrial membrane which triggers the release of cytochrome *c* from the mitochondrial intermembrane space. In the cytoplasm, cytochrome *c* activates the adaptor protein Apaf-1 by facilitating its heptamerization, thereby forming a signaling complex called the apoptosome. The apoptosome then recruits and activates caspase-9. There are two classes of caspases that lead to the dismantling of cellular components in both pathways: initiator caspases and effector (executioner) caspases. Caspases-8, -9, and -10 serve as the initiator caspases, as they are activated first in response to a pro-apoptotic stimulus through the DISC or the apoptosome (Figure 2). The initiator caspases then gain the ability to cleave and functionally activate downstream effector caspases, such as caspases-3 and -7. These activated effectors then cleave an array of cellular protein substrates, which leads to apoptotic death. Importantly, caspases-8 and -10 can cleave the BH3-only protein BID to generate its active form, tBID, which in turn promotes mitochondrial cytochrome *c* release. Therefore, BID can play a role in both the extrinsic and intrinsic pathways. In contrast to caspases-8, -9, and -10, caspase-2 is an initiator caspase whose mechanisms and functions remain less understood even though it is the most evolutionally conserved, with structural similarities to *C. elegans* and *Drosophila* caspases, CED-3 and DRONC, respectively. Caspase-2 is activated by another death signaling complex called the PIDDosome, which consists of the adaptor proteins PIDD and RAIDD. The formation of the PIDDosome itself is fully independent of cell death receptors or cytochrome *c*. Although it is known that the PIDDosome can be induced in response to DNA damage (see [7] for caspase-2 review), the mechanism of its formation and caspase-2 activation remains elusive. The substrates of caspase-2 have also been a subject of much debate. Except for BID, very few proteins are known to be cleaved by caspase-2 [7].

## 3. Knocking Out Pro-Survival *Bcl-2* Family Genes

The first clue of the involvement of the BCL-2 family proteins in female germ cell death came from the early studies of pro-survival *Bcl-2* gene knockout mice, where it was shown that whole-body knockout resulted in the loss of primordial follicles [8]. Subsequently, two groups independently generated transgenic mouse lines and demonstrated that overexpression of *Bcl-2* in oocytes could lead to an increase in the primordial follicle pool or the resistance of oocytes to apoptosis [9,10]. Interestingly, however, a more recent study did not observe a significant difference in the primordial follicle pool of the neonatal mouse ovary upon oocyte-specific knockout or overexpression of *Bcl-2* [11]. This discrepancy suggests that BCL-2 may play a role in the prevention of follicle atresia after puberty, but not in the survival of oocytes in the fetal or neonatal stage. Alternatively, it may be ascribed to the presence of multiple BCL-2 family proteins with redundant functions in mouse oocytes as we discuss below. (See Table 1).

Unlike *Bcl-2*, *Bcl-X_L_* null embryos die at 13 d.p.c. (days post coitum) [43]. However, a conditional depletion study where the expression of both *Bcl-X_L_ and Bcl-X_S_* isoforms was significantly diminished by targeting the *Bcl-X* promoter has shown that the loss of *Bcl-X* leads to a marked reduction in germ cell numbers in fetal ovaries, from 13.5 to 15.5 d.p.c. [12] (note: BCL-X_S_ is a short isoform and pro-apoptotic). Interestingly, using this mouse model, the same group later showed that there were no differences in the number of primordial, primary, and antral follicles in the ovary of *Bcl-X*-depleted adult mice [13]. Moreover, *Bcl-X*-depleted female mice were fertile despite a smaller litter size compared to wild-type females [13]. These results suggest that while BCL-X_L_ plays a role in the protection of embryonic germ cells, it is dispensable for follicular development and female fertility.

The pro-survival MCL-1 is structurally somewhat distinct from BCL-2 and BCL-X_L_, although it contains BH domains 1–4 and a C-terminal transmembrane domain like the other pro-survival BCL-2 family members [4]. Thus, unlike BCL-2 and BCL-X_L_, MCL-1 does not interact with the BH3-only protein BAD, but it is capable of binding to NOXA. *Mcl-1* knockout is embryonically lethal, where *Mcl-1* null embryos die at the peri-implantation stage [44]. However, various tissue-specific conditional *Mcl-1* knockout mice have been generated and are viable. Interestingly, oocyte-specific *Mcl-1* gene knockout results in a significant reduction in the number of ovarian follicles, including primordial follicles [14]. Consequently, the mice experienced poor ovulation rates at 3 months and older, although oocyte-specific *Mcl-1* knockout did not completely abolish ovulatory capacity [14]. These results suggest that MCL-1 is required for postnatal maintenance of the follicular pool.

BCL-w (also known as Bcl2l2 or Kiaa0271), Diva (also known as Bcl2l10, Boo, or BCL-b), and A-1 (also known as Bcl2a1a or BFL-1) are additional pro-survival BCL-2 family proteins whose functional significance in development and human disease is less characterized. *Bcl-w* gene knockout mice were viable and showed no phenotypes except for failed spermatogenesis [15]. Likewise, *Diva* null-mice exhibited normal ovarian histology [16], and its depletion did not affect the sensitivity to radiation-induced apoptosis in the ovary compared to wild-type mice [16]. Moreover, a transgenic mouse line that lacks all A-1 isoforms appeared outwardly normal and showed only minor defects in immune cells [17]. These results suggest that BCL-w, Diva, and A-1 are either dispensable for ovarian functions and oocyte development, or their loss can be compensated for by other pro-survival BCL-2 family proteins.

## 4. Knocking Out Pro-Apoptotic *Bcl-2* Family Genes

Knockout mice targeting one or more genes encoding BH3-only proteins (BAD, BIK, BID, PUMA, BIM, BMF, NOXA, and HRK) have been generated, but none of them displayed any differences in ovarian functions or female fertility except for *Bmf*-deficient mice [27] (see Table 1). Given their overlapping role in activating the multi-BH domain proteins, it is not surprising that single knockout does not lead to a prominent phenotype, especially under non-stressed conditions. However, the deletion of the multi-BH domain proteins provides interesting insights into the distinct and overlapping roles of these proteins in the regulation of oocyte apoptosis. For example, *Bak* knockout mice are healthy and fertile [30], whereas *Bax* knockout mice exhibit lymphoid hyperplasia [45]. Moreover, *Bax*-null male mice are infertile due to defective spermatogenesis [45]. Importantly, *Bax* knockout mice display a three-fold increase in the number of primordial follicles at 42 days old compared to that in aged-matched wild-type controls, and this follicle surplus appears to contribute to the extended ovarian lifespan in *Bax* deficient females [32]. *Bax/Bak* double knockout mice display severe developmental abnormalities which were not observed in *Bax* or *Bak* single knockout mice, including frequent death in the pre- and perinatal periods, as well as the presence of interdigital webs and imperforate vagina in live offspring [30]. *Bax/Bak* double knockout also confers marked resistance in many cell types to various intrinsic apoptotic stimuli which activate the intrinsic pathway [30,46]. *Bax/Bak* double knockout males, however, remain infertile due to failed spermatogenesis as seen in *Bax* single knockout mice [30]. Whether co-depletion of *Bax* and *Bak* would further increase the ovarian reserve compared to *Bax* single knockout, remains to be determined. In this regard, it is noteworthy that the reduction in the ovarian reserve in *Mcl-1* [14] and *Bcl-X* [12] knockout mice can be rescued by concomitant deletion of the *Bax* gene. Concurrent deletion of *Bax* also restores normal ovulation rates and improves the breeding performance of oocyte-specific *Mcl-1* knockout mice [14]. These results suggest that it may be BAX, not BAK, that plays a major role in the regulation of oocyte apoptosis, at least in mice.

While BAX and BAK are often essential for the mitochondrial apoptosis pathway in human disease and development, the role of BOK remains less studied. *Bok* was initially discovered through a yeast-two-hybrid screening with a rat ovarian cDNA library using *Mcl-1* as bait [47]. As expected, BOK is highly expressed in the ovary, especially in oocytes and granulosa cells [47,48]. However, whole body knockout did not result in any prominent phenotypes in mice [33]. Interestingly, *Bok/Bax*, but not *Bok/Bak*, double knockout females displayed an increase in the number of ovarian follicles at one year of age, exacerbating the phenotype caused by loss of *Bax* [31]. Since this abundance of ovarian follicles was not seen at 14 weeks of age [31], it is unlikely that the double knockout females were supplied with excess primordial follicles at birth. Interestingly, except for the ovarian phenotype, the deletion of *Bok* did not significantly worsen the phenotypes of *Bax* or *Bak* single knockout mice, respectively [31], suggesting that BAX and BOK have an overlapping role in oocyte survival and the maintenance of the ovarian pool in adults.

## 5. Caspase Knockout Mice

Caspases are the ultimate executioners of apoptosis. However, it remains unclear which one(s), especially effector caspases, carry out this process in oocytes, which may be challenging to uncover. This can be attributed to potential compensatory mechanisms to offset the loss of a caspase gene by the upregulation of another [49], along with their overlapping roles. In general, caspase-3 is the most abundant effector caspase and plays a critical role in the induction of apoptosis in many settings. However, *caspase-3* gene inactivation had no effect on the number of ovarian follicles [34]. It is possible that another major executioner, such as caspase-7, can compensate for its absence. Likewise, *caspase-7* knockout mice display a healthy appearance and normal tissue morphology [35]. Although the fetuses at 20 d.p.c. appear normal, mice lacking both *caspases-3* and *-7* die soon after birth [35]. Unfortunately, to our best knowledge, the analysis of the ovarian reserve in *caspases-3/7* double knockout mice has not been reported.

The role of caspase-2 is somewhat mysterious because its activation mechanism and substrates remain elusive. As mentioned earlier, the formation of the PIDDosome, the caspase-2-activating platform, can be promoted upon DNA damage. In fact, oocytes collected from *caspase-2* knockout mice display significant resistance to the DNA-damaging agent doxorubicin, compared to oocytes from wild-type mice [36]. Although it is an in vitro study with isolated oocytes, this result suggests that caspase-2 may be involved in oocyte apoptosis induced by chemotherapeutic drugs. Interestingly, female mice lacking *caspase-2* also have a much larger pool of primordial follicles at postnatal day (PND) 4 [36], suggesting that caspase-2 may play a key role in the death of female germ cells that occurs at the perinatal period through PND 3. In contrast, *caspase-11* (= *caspase-4*) knockout leads to the marked reduction of primordial follicles at PND 4, which is likely secondary to impaired cytokine production [37]. Interestingly, the death of oocytes caused by *caspase-11* deficiency can be rescued by the co-deletion of *caspase-2* [37].

## 6. The Extrinsic Pathway

Since an early study demonstrated that the injection of an agonistic anti-Fas antibody triggered follicular atresia in mice [39], it has been known that the extrinsic pathway plays a role in oocyte development and maintenance. Adult *lpr/lpr* mice, which carry a mutation in the *Fas* gene (resulting in reduced *Fas* expression), display an increase in ovarian follicles, particularly secondary follicles, compared to wild-type mice [39]. Later, another study using *Fas* knockout mice also showed that *Fas*-deficient mice had more ovarian follicles, compared to wild-type mice at PNDs 2 and 14 [40]. It should be noted, however, that the cell death receptor Fas is expressed in granulosa cells of secondary and antral follicles, but not in oocytes [39]. Thus, these results suggest that the Fas-mediated extrinsic apoptosis pathway regulates follicular development by controlling granulosa cell death.

TNFα is a cytokine with a wide variety of roles, ranging from serving as a cell survival ligand to being an activator of the extrinsic apoptosis pathway. Unlike Fas, TNFα is expressed in both oocytes and granulosa cells [50,51]. At PND 4, TNFα knockout females displayed a two-fold increase in primordial follicles compared to wild-type females [41]. Moreover, at one year of age, *Tnfα* knockout females maintained a larger follicular pool than wild-type females, and their litter size was significantly, and consistently, larger than that of wild-type females during a 12-month breeding period [41]. Whether the *Tnfα* knockout phenotype can be ascribed to the loss of the extrinsic pathway in oocytes, granulosa cells, or both, remains to be determined. TNFR1 and TNFR2, the two receptors shared by TNFα, are expressed in oocytes [42]. The analysis of *Tnfr1* and *Tnfr2* single knockout mice showed that the ovary in *Tnfr2* knockout females at PND 7 had more growing follicles compared to the ovary in wild-type mice [42]. Moreover, when analyzed at PND 80, the number of primordial follicles was significantly greater in the ovary in *Tnfr2* knockout females compared with the ovary in wild-type females [42]. Interestingly, *Tnfr1* knockout females did not show such a phenotype [42].

Importantly, it is not known which caspase(s) is responsible for the induction of the extrinsic pathway in the ovary. In this regard, multiple studies have reported that caspase-2 can be activated in a PIDDosome-independent manner, in particular, through the DISC, including the TNFR complex [7]. Given the similarity between the ovarian phenotypes observed in *caspase-2* knockout mice and *Tnfα* (*Fas* or *Tnfr2*) knockout mice, it is tempting to speculate that the induction of the extrinsic pathway may lead to the activation of caspase-2 in the ovary.

The oocyte phenotypes of transgenic mouse models related to the apoptosis pathways are summarized in Table 1.

## 7. p53 Family Proteins

In mammalian cells, the p53 family of transcription factors plays a key role in the regulation of many cellular functions including cell death, cell-cycle arrest, and DNA repair. This family largely consists of p53, p63, and p73 which all share a structurally homologous DNA binding domain, transactivation domain, and tetramerization domain [52]. In addition, the p53 family proteins share some of the same target genes, such as pro-apoptotic *Puma* and *Noxa* (see below) [53,54].

**Figure 3:**
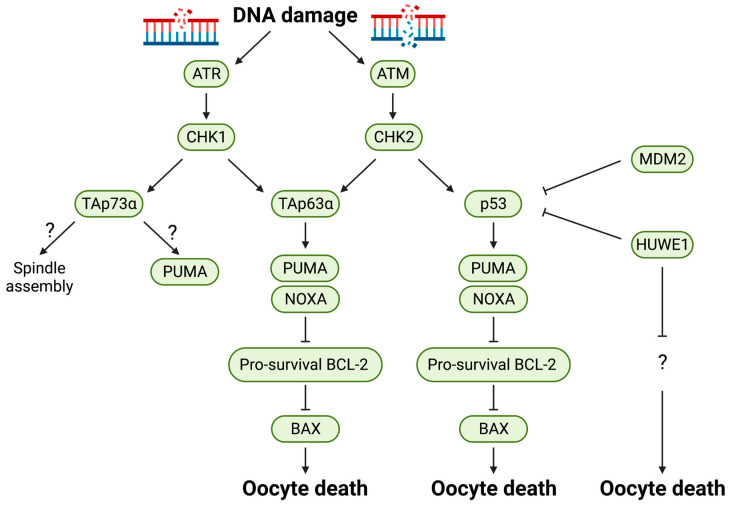
The DNA damage response pathway. In mouse oocytes, DNA single-strand and double-strand breaks trigger the activation of ATR or ATM, respectively. Once activated, ATR and ATM phosphorylate and activate their downstream kinases CHK1 and CHK2, respectively. Please see the text for more detail.

Among the p53 family proteins, p53 is the most studied family member since it is frequently inactivated in human cancer, and because its genetic inactivation promotes spontaneous carcinogenesis in mice [55]. Following cytotoxic or genotoxic stress, such as DNA damage, p53 is activated and induces an array of genes that arrest the cell cycle. However, if the damage persists or is irreparable, it promotes apoptosis by inducing genes such as *Bax*, *Puma*, *Noxa*, and *Apaf-1* [56]. Moreover, p53 is regulated at multiple levels. While post-translational modifications (e.g., phosphorylation) play an important role in p53 activation, p53 protein levels are also tightly regulated by multiple ubiquitin E3 ligases, including MDM2 and MDMX [52]. Conditional deletion of *Mdm2*, but not *Mdmx*, in mouse oocytes leads to female infertility due to a reduction in the number of healthy follicles and increased follicular atresia [57]. Importantly, co-deletion of *p53* can restore female fertility [57], indicating that the induction of p53-target genes is sufficient to cause oocyte apoptosis. HUWE1 (also known as ARF-BP1 or MULE) is another ubiquitin E3 ligase that targets p53 for proteasomal degradation [58]. Recently, we demonstrated in mice that oocyte-specific *Huwe1* knockout kills oocytes at the GV stage, resulting in complete infertility [59]. Interestingly, unlike *Mdm2* knockout [57], concomitant p53 deletion does not rescue this phenotype [59]. The specific substrate(s) of HUWE1 that regulates oocyte death remains to be identified (Figure 3).

p63 was first discovered as a p53 homolog, nearly two decades after the discovery of p53 [60,61,62]. In the context of cancer, p53 is one of the essential tumor suppressors and is often mutated or deleted in cancer cells, but p63 mutation or deletion is rare [63]. Accumulating evidence suggests that p63 has roles in development. Unlike p53 knockout mice, p63 knockout mice die during embryogenesis due to abnormal limb and epithelium formation [64,65]. In addition to these roles, p63 also functions in oocytes as a “guardian of the genome”. While p63 maintains genomic integrity under non-stressed conditions, it also triggers oocyte apoptosis in response to genotoxic stress [66,67]. Moreover, p63 has an alternative promoter and undergoes alternative splicing, resulting in at least six isoforms. The predominant form in mammalian primordial follicle oocytes is TAp63α [66]. Importantly, TAp63α-null oocytes are resistant to apoptosis induced by γ-irradiation, while p53-null oocytes die within 2 days [66], indicating that TAp63α, but not p53, is the main player in oocytes for inducing apoptosis in response to DNA damage. It should be noted, however, that although TAp63α-null oocytes show marked resistance to γ-irradiation initially, they eventually die [68]. Moreover, *p53/TAp63α* double knockout oocytes display resistance to γ-irradiation for an extended period of time [68], suggesting that both p53 and p63 may contribute to the DNA damage response where p63 plays a more prominent role. What downstream effector(s) causes apoptosis in a p63-dependent manner following DNA damage? It was demonstrated that primordial follicle oocytes from mice deficient for the BH3-only protein PUMA are strikingly resistant to γ-irradiation [22]. Moreover, although oocytes from mice deficient in NOXA, another BH3-only protein, do not display any phenotypes by themselves, the depletion of *Noxa* further augments the irradiation-resistant phenotype of *Puma*-null oocytes [22], indicating that both *Puma* and *Noxa*, common target genes of p53 and p63, are responsible for DNA damage-induced oocyte apoptosis (Figure 3). Importantly, while oocytes from p53 deficient mice still express PUMA and NOXA, oocytes from TAp63 deficient mice do not [22]. This supports the idea that p63 may play a more dominant role in inducing these two BH3-only proteins in oocytes. The essential role of PUMA and NOXA in DNA damage-induced oocyte apoptosis was also shown by a more recent study in which oocytes with defective recombination repair (*Dmc1^−/−^* and *Msh5^−/−^*) were employed [23]. Oocytes in *Dmc1^−/−^* and *Msh5^−/−^* mice experience persistent DNA damage due to impaired meiotic recombination, resulting in death at/before the primordial follicle stage. Interestingly, co-deletion of *Puma* and *Noxa* could rescue oocyte loss in *Dmc1^−/−^* and *Msh5^−/−^* mice [23]. More importantly, the same study also demonstrated that much like *Puma/Noxa* double knockout, *Bax* single knockout alone can also protect oocytes from death in *Dmc1^−/−^* and *Msh5^−/−^* mice, as well as from irradiation-induced apoptosis [23]. Therefore, it is strongly suggested that DNA damage-induced oocyte apoptosis is mediated by the induction of PUMA and NOXA, followed by BAX activation (Figure 3 and Table 2).

DNA damage leads to the activation of ATR and ATM kinases, which activate Checkpoint kinase 1 (CHK1, CHEK1) and Checkpoint kinase 2 (CHK2, CHEK2), respectively (Figure 3). The global knockout of *Atr* leads to embryonic lethality before 7.5 d.p.c. due to chromosomal fragmentation [72], whereas *Atm* deficiency results in genome instability and enhanced radiosensitivity [70,71,78]. Moreover, *Atm* deficiency also causes significant apoptosis in germ cells due to meiotic defects, which leads to infertility in both male and female mice [70,71]. Interestingly, oocyte apoptosis in *Atm* knockout mice was not rescued by concomitant deletion of *caspase-2* or *Bax* [37], suggesting that there may be a yet-to-be-identified apoptosis pathway that operates under severe meiotic defects in the fetal ovary. *Chk1* deficiency causes p53-independent apoptosis at the blastocyst stage due to DNA replication defects [79]. Interestingly, a transgenic mouse line that expresses an extra copy of *Chk1* (*SuperChk1* or *sChk1*) displays an increase in the ovarian reserve at birth and long-term maintenance of the follicular pool [69]. This suggests that even under non-stressed conditions, chronic DNA damage constantly occurs in oocytes before and after birth, leading to the depletion of the ovarian reserve that may be protected by the upregulation of the DNA repair pathway [69]. In contrast to *Chk1* deficiency, *Chk2* knockout mice develop normally and are viable. Interestingly, aged *Chk2* knockout female mice show a larger follicular pool at an older age (13.5 months) compared to wild-type controls, which results in delayed reproductive senescence [69]. However, there were no differences in the ovarian reserve in young mice (1.5 months) [69], suggesting that CHK2 only plays a role in the depletion of the ovarian reserve after puberty. More importantly, the gene knockout of CHK2, which can phosphorylate and activate both p53 and p63 (Figure 3), recapitulates the phenotype of *p53/TAp63α* double knockout oocytes that display marked resistance to DNA damage [68] (Table 2). This strongly suggests that oocyte apoptosis induced by irradiation is mediated by CHK2 phosphorylation of p53 and TAp63α.

Compared to p53 and p63, the role of p73 in oocyte apoptosis remains poorly understood. The most predominant isoform of p73 in oocytes is TAp73α [73]. *Trp73* knockout females (in which all p73 isoforms were removed) [76] and *TAp73* knockout females (in which only TAp73 isoforms were deleted) [75] showed that they are infertile partially because ovulated oocytes are trapped under the ovarian bursa, thus not being released into the fallopian tubes. Nevertheless, *TAp73*-deficient ovaries contain fewer primordial and primary follicles compared to wild-type ovaries [75]. Moreover, *TAp73*-deficient oocytes display severe abnormalities in spindle assembly, resulting in embryonic death at the blastocyst stage following in vitro fertilization with wild-type sperm [75]. These results suggest that, unlike p53 and p63, p73 plays a role in the formation of spindle assembly rather than the induction of apoptosis. Nevertheless, a recent ex vivo study in ovarian culture demonstrated that oocyte-specific *Trp73* deficiency partially protects oocytes from cisplatin-induced apoptosis, but not from X-ray-induced apoptosis [77]. As p73 can induce apoptosis in cancer cells by inducing *Puma* [80], how p73 plays its role in oocyte quality control awaits further investigation (Figure 3).

## 8. Conclusions

Females are born with a finite number of oocytes. However, the vast majority of them die by apoptosis before leaving the ovary. Because oocytes are single cells, the apoptotic machinery must carefully decide cell fate. Yet, the mechanism that governs this decision in vivo remains elusive. Oocytes are a difficult cell type to study since unlike cell lines, oocytes only function within the ovary. Over the last twenty-five years, a number of gene knockout mice have been generated, providing us with a window of opportunity to genetically dissect the signaling pathway. Ultimately, this collection of in vivo evidence should help us to understand how oocyte apoptosis might be regulated in human ovaries.

## Figures and Tables

**Table 1:** Gene knockout mice related to the apoptosis pathway.

Genes	Type of Knockout		Phenotypes	References
*Bcl-2*	Global KO	Pro-survival BCL-2 family member	Decrease in the number of primordial follicles in ovaries at PND 42	[8]
	Global transgenic overexpression		Increase in the number of primordial follicles at PND 8 but not retained in adulthood	[9]
	Oocyte-specific overexpression		Decrease in follicular atresia at PND 42 but no significant phenotypic differences in neonatal ovaries between PNDs 1 and 7	[10,11]
*Bcl-X* (*Bcl2l1*)	Reduction in Bcl-X (both isoforms: *Bcl-X_L_* and *Bcl-X*_s_) gene expression	Pro-survival BCL-2 family member	Drastic decrease in primordial follicle numbers at PND 19 but no significant phenotypic differences in ovaries in later study using conditional knockout	[12,13]
*Mcl-1*	Oocyte-specific KO	Pro-survival BCL-2 family member	Decrease in primordial oocyte reserve at PND 7	[14]
			Decrease in the numbers of primordial follicles, primary follicles and secondary follicles at 3 months which can be rescued by co-deletion of *Bax*	
*Bcl-w* (*Bcl2l2*, *Kiaa0271*)	Global KO	Pro-survival BCL-2 family member	Fertile and no abnormalities reported in the ovary	[15]
*Diva* (*Bcl2l10*, *Bcl-b*, *Boo*)	Global KO	Pro-survival BCL-2 family member	Fertile and no abnormalities reported in the ovary	[16]
*A-1* (*Bcl2a1a*, *Bfl-1*)	Global KO	Pro-survival BCL-2 family member	Fertile and no abnormalities reported in the ovary	[17]
*Bad* (*Bbc6*)	Global KO	Pro-apoptotic BH3-only protein	Ovarian phenotypes not reported	[18]
*Bik* (*Biklk*, *Blk*, *Nbk*)	Global KO	Pro-apoptotic BH3-only protein	Fertile and no abnormalities reported in the ovary	[19]
*Bid*	Global KO	Pro-apoptotic BH3-only protein	Ovarian phenotypes not reported	[20,21]
*Puma* (*Bbc3*)	Global KO	Pro-apoptotic BH3-only protein	No ovarian phenotype under non-stressed conditions	[22]
			Primordial follicle oocytes are resistant to irradiation-induced apoptosis at PND 5	
*Noxa* (*Pmaip1*)	Global KO	Pro-apoptotic BH3-only protein	No significant differences in ovarian morphology	[22]
*Puma/Noxa*	Global DKO	Pro-apoptotic BH3-only proteins	*Puma/Noxa* DKO oocytes are more resistant to DNA damage-induced death than *Puma* single KO oocytes	[22]
			Protected oocyte death in *Dmc1* and *Msh5*-nulls (oocytes with defective recombination repair)	[23]
*Bim* (*Bcl2l11*)	Global KO	Pro-apoptotic BH3-only protein	Fertile and no abnormalities reported in the ovary	[24]
*Bim/Bad*	Global DKO	Pro-apoptotic BH3-only proteins	Fertile and no abnormalities reported in the ovary	[25]
*Bim/Bik*	Global DKO	Pro-apoptotic BH3-only proteins	Fertile and no abnormalities reported in the ovary	[24]
*Bim/Bid*	Global DKO	Pro-apoptotic BH3-only proteins	Ovarian phenotypes not reported	[26]
*Bid/Bim/Puma*	Global TKO	Pro-apoptotic BH3-only proteins	Ovarian phenotypes not reported	[26]
*Bmf*	Global KO	Pro-apoptotic BH3-only protein	Increase in follicles at PNDs 100, 200, 300, and 400	[27]
			Fertile and no abnormalities reported in the ovary	[28]
*Hrk* (*Bid3, Dp5*)	Global KO	Pro-apoptotic BH3-only protein	Fertile and no abnormalities reported in the ovary	[29]
*Bak* (*Bak1*)	Global KO	Pro-apoptotic BCL2 family member	Fertile and no abnormalities reported in the ovary	[30,31]
*Bax*	Global KO	Pro-apoptotic BCL2 family member	Three times as many primordial follicles at PND 42 and reduced follicular atresia (granulosa cell death) induced by apoptosis. No increase observed in primordial follicles in neonatal ovaries	[32]
	Global KO		Fertile and no abnormalities reported in the ovary	[31]
	Global KO		Protected oocytes from irradiation-induced death and the lack of *Dmc1* and *Msh5*-nulls (oocytes with defective recombination repair)	[23]
*Bok* (*Mtd*)	Global KO	Pro-apoptotic BCL2 family member	Fertile and no abnormalities reported in the ovary	[31,33]
*Bok/Bak*	Global DKO	Pro-apoptotic BCL2 family members	Fertile and no abnormalities reported in the ovary	[31]
*Bok/Bax*	Global DKO	Pro-apoptotic BCL2 family members	Aged (1-year-old) *Bok/Bax* DKO females had excess follicles at almost all developmental stages, exacerbating the phenotype caused by *Bax* single KO	[31]
*Caspase-3* (*Casp3*, *Cpp32*)	Global KO	Protease	No significant differences in ovarian morphology	[34]
*Caspase-7* (*Casp7*, *Lice2*, *Mch3*)	Global KO	Protease	No significant differences in ovarian morphology	[35]
*Caspase-2* (*Casp2*, *Ich1*, *Nedd2*)	Global KO	Protease	Increase in the number of primordial follicles at PND 4	[36]
			Resistance to apoptosis induced by doxorubicin in young adult mice	
*Caspase-11* (*Casp4*, *Casp11*, *Caspl*, *Ich3*)	Global KO	Protease	Severely diminished primordial follicle pool at PND 4 which can be rescued by *Caspase-2* KO	[37]
*Caspase-9* (*Casp9*, *Mch6*)	Global KO	Protease	At 19.5 d.p.c., when the majority of oocytes complete homologous recombination, the total number of germ cells was noticeably larger in *Caspase-9* KO embryos.	[38]
*Fas* (*Apt1*, *Tnfrsf6*)	Global KO	Cell death receptor	Increase in secondary follicles	[39]
			Increase in germ cells/oocytes in prenatal and PND 2 to 14 ovaries	[40]
*Tnfa* (*Tnf*, *Tnfsf2*)	Global KO	Cell death ligand	Increase in the number of total follicles from PND 4 to 90	[41]
*Tnfr1* (*Tnfrsf1a*)	Global KO	Cell death receptor	No significant differences in ovarian morphology	[42]
*Tnfr2* (*Tnfrsf1b*)	Global KO	Cell death receptor	Increase in the number of primary follicles at PND 7 and primordial, primary and preantral follicles at PND 80	[42]

DKO = double knockout; TKO = triple knockout; PND = postnatal day; d.p.c. = days post coitum.

**Table 2:** Gene knockout mice related to the DNA damage pathway.

Genes	Type of Mouse Models	Protein Functions	Oocyte Phenotypes	References
*Chk1* (*Chek1*)	Conditional aneuploid mutant with 3 copies of *Chk1*	Kinase	Increase in primordial, primary and antral follicles in 1.5 months old mice as well as primordial and antral follicles in aged mice	[69]
*Chk2* (*Chek2*)	Oocyte-specific KO	Kinase	No difference in 1.5 month old mice and increase in primordial, primary, secondary and antral follicles in aged mice	[69]
	Global KO		Rescued the infertility of *Trip13^Gt/Gt^* (DNA repair deficient) and irradiated females	[68]
*Atm*	Global KO	Kinase	Infertile and decrease in oocyte reserve in adult females	[70,71]
*Atr* (*Kiaa4069*)	Global KO	Kinase	Embryonic lethality due to DNA fragmentation between blastocyst stage and 7.5 d.p.c	[72]
*Trp53* (*Tp53*, *P53*)	Global KO	Transcription factor	Partially rescued the *Trip13^Gt/Gt^* (DNA repair deficient) oocytes	[68]
*Trp63* (*Tp63*, *P63*)	Oocyte-specific KO	Transcription factor	Rescued from cisplatin-induced cell death of primordial follicles in ovary (PND 5) after 4 days of culture	[73]
	Global KO		Rescued from irradiation-induced cell death of primordial follicles in ovary (18.5 d.p.c)	[74]
*TAp63*	Global KO	Transcription factor	Primordial follicles protected from irradiation induced apoptosis at PND 5 in *TAp63* KO	[66]
*TAp73*	Global KO	Transcription factor	Decrease in oocyte reserve and infertility	[75]
*Trp73*	Global KO	Transcription factor	Infertile but no abnormality in oocytes	[76]
	Oocyte-specific KO	Transcription factor	Partially rescued from cisplatin-induced cell death of primordial follicles in ovary (PND 5), but not from X-ray induced apoptosis	[77]
*Mdm2*	Oocyte-specific KO	Ubiquitin E3 Ligase	Infertile and decrease in healthy secondary and tertiary follicles and increase in atretic primary, secondary follicle population at 5–6 weeks of age. Fertility was restored in *Mdm2/p53* DKO	[57]
*Huwe1* (*Kiaa0312*, *Ureb1*)	Oocyte-specific KO	Ubiquitin E3 Ligase	Infertile and less follicles present at 4 weeks of age. Fertility was not restored in *Huwe1/p53* DKO	[59]

DKO = double knockout; PND = postnatal day; d.p.c. = days post coitum.

## Data Availability

Not applicable.
